# Brain targeted lactoferrin coated lipid nanocapsules for the combined effects of apocynin and lavender essential oil in PTZ induced seizures

**DOI:** 10.1007/s13346-024-01610-0

**Published:** 2024-05-31

**Authors:** Julie R. Youssef, Nabila A. Boraie, Fatma A. Ismail, Basant A. Bakr, Eman A. Allam, Riham M. El-Moslemany

**Affiliations:** 1 Department of Pharmaceutics, Faculty of Pharmacy, Alexandria University, 1 Khartoum Square, Azarita, Messalla Post Office, P.O. Box 21521 Alexandria, Egypt; 2 Department of Zoology, Faculty of Science, Alexandria University, Alexandria, 21523 Egypt; 3 Department of Medical Physiology, Faculty of Medicine, Alexandria University, Alexandria, 21131 Egypt

**Keywords:** NADPH-oxidase inhibitor, Essential oil, Epilepsy, Subcutaneous, Bioavailability, Log BB

## Abstract

Apocynin (APO) is a plant derived antioxidant exerting specific NADPH oxidase inhibitory action substantiating its neuroprotective effects in various CNS disorders, including epilepsy. Due to rapid elimination and poor bioavailability, treatment with APO is challenging. Correspondingly, novel APO-loaded lipid nanocapsules (APO-LNC) were formulated and coated with lactoferrin (LF-APO-LNC) to improve br ain targetability and prolong residence time. Lavender oil (LAV) was incorporated into LNC as a bioactive ingredient to act synergistically with APO in alleviating pentylenetetrazol (PTZ)-induced seizures. The optimized LF-APO-LAV/LNC showed a particle size 59.7 ± 4.5 nm with narrow distribution and 6.07 ± 1.6mV zeta potential) with high entrapment efficiency 92 ± 2.4% and sustained release (35% in 72 h). Following subcutaneous administration, LF-APO-LAV/LNC brought about ⁓twofold increase in plasma AUC and MRT compared to APO. A Log BB value of 0.2 ± 0.14 at 90 min reflects increased brain accumulation. In a PTZ-induced seizures rat model, LF-APO-LAV/LNC showed a Modified Racine score of 0.67 ± 0.47 with a significant increase in seizures latency and decrease in duration. Moreover, oxidant/antioxidant capacity and inflammatory markers levels in brain tissue were significantly improved. Histopathological and immunohistochemical assessment of brain tissue sections further supported these findings. The results suggest APO/LAV combination in LF-coated LNC as a promising approach to counteract seizures.

**Graphical Abstract:**

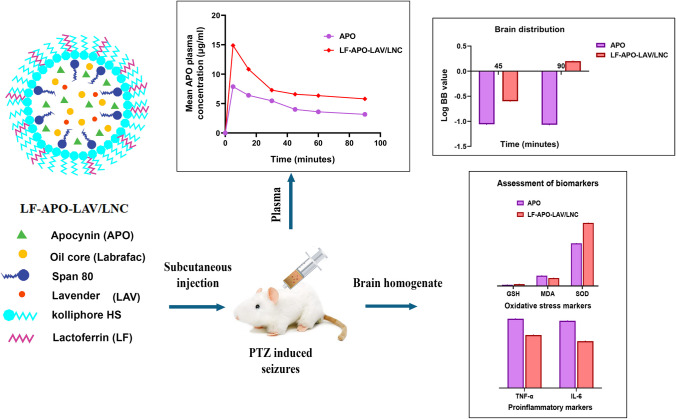

## Introduction

Epilepsy is one of the most common neurological disorders, affecting over 1% of the total world population [1]. It is featured by unusual synchronous neuronal excitability of the cerebral neurons generating spontaneous and repetitive unaggravated seizures [2]. Cerebral trauma, tumors, infections and immunological disorders in addition to hippocampal sclerosis and cerebrovascular disorders are all causatives of epileptic seizures [3]. Seizures are accompanied by major psychological, physical, and socioeconomic consequences [4, 5]. Although the molecular mechanism underlying epileptic seizures is yet vague, it is conjectured that neuroinflammation, neuronal apoptosis and oxidative stress contribute to aberrant neuronal injury mediating epileptic seizures onset [6].

The aim of epilepsy control is to reduce the frequency and severity of seizures while keeping minimum toxicity to the body. Despite the increasing number of conventional antiepileptic drugs, prominent side effects are limiting their use [7]. Also, a major obstacle facing anti-epileptic drugs is the inability to cross the blood brain barrier (BBB) [8] which is composed of tightly connected cells allowing limited access of molecules to the brain. Owing to these tight junctions about 98% of all small molecules and approximately 100% of large molecules are unable to reach the neural tissues [9]. Moreover, drug delivery across the diseased BBB is more complex than across the normal brain as alterations in this unique barrier affect drugs’ brain accumulation and intracranial distribution [10]. The BBB microvascular dysfunction associated with epileptogenesis and seizure disorder [11] endows therapeutic agents entrapment in pathologically altered and enlarged perivascular spaces, preventing them from reaching distant neural targets [12]. Furthermore, epileptic patients show drug resistance due to up-regulation of the drug efflux transporter; P-glycoprotein (P-gp) [13, 14].

Amongst the myriad of approaches applied to improve drug delivery to the brain, nanotechnology gained an increasing attention making diversly new biological discoveries feasible through modulating the physicochemical, physiological, and bioactive features of drugs. Furthermore, nano scaled carriers grant selective and targeted drug delivery to the brain reducing hazardous effects on healthy tissue [15]. Previous studies clarified the therapeutic antiepileptic effect of nanocarriers as liposomes and polymeric or lipid nanoparticles in animal models of epilepsy [16, 17]. Fine-tuning of nanosystem structure, composition and physicochemical properties affect the cellular uptake and response to different nanoparticles [18].

Lipid nanocapsules (LNC) are a monodisperse system composed of an oily liquid triglyceride core surrounded by a polyethoxylated surfactant rigid membrane with a size range 20–100 nm [19]. LNC offer the advantage of encapsulating hydrophobic active ingredients with high entrapment efficiency and sustained drug release [20]. In addition, the PEG external casing allows for LNC prolonged circulation and ability to suppress P-gp efflux pump thus promoting an increase in the drug concentration reaching its specific target [21] which is expected to display a crucial role in increasing drug delivery to the brain by down regulation of these multi drug resistant transporters [22]. Also, due to their formulation from GRAS ingredients, in vivo studies authenticated safety and suitability of LNC in drug delivery to the CNS [23]. Interestingly, LNC surface is amenable to modification with a variety of biochemically active groups with definable active targetability [15].

For enhanced drug delivery to the brain, lactoferrin (LF) receptors were shown to play a pivotal role. Lactoferrin (LF) is an iron binding glycoprotein, with multiple biological activities such as immunomodulatory, antioxidant, anti-bacterial and antiviral actions [24–26]. LF receptors are immensely expressed in brain microvasculature and neurons [27] facilitating its transport across the BBB both in vitro and in vivo. LF was previously depicted as neuroprotective agent used in controlling neurodegenerative disorders [28]. Applying LF coating to various lipid nanoparticles succeeded in enhancing brain targetability and hence efficacy via targeting LF receptors. Chen et al. showed improved doxorubicin efficacy following LF coating of pro cationic liposomes in gliomas [29]. Also, biocompatible LF decorated nanostructured lipid carriers encapsulating nimodipine proved enhanced activity for cerebral vascular stroke treatment [30].

Another promising approach to overcome the drawbacks of conventional antiepileptic drugs is the use of herbal drugs. Nowadays rigorous research is focusing on the alternative use of phytomedicine in the management of seizures [31]. Among the bioactive phytochemicals investigated, apocynin (APO) (4-hydroxy-3-methoxyacetophenone) obtained from the roots of *Apocynum cannabinum* [32]; revealed neuroprotective effects in several CNS disorders [33–35]. Recently, Gagandeep et al. [2] studied the effect of APO in a pentylenetetrazol (PTZ) kindling epilepsy model. APO acts as an inhibitor of NADPH-oxidase enzyme complex interfering with the assembly of cytosolic components [35] through preventing the shift of p47-phox protein sub-units from the cytoplasm to the cell membrane [36]. NADPH-oxidase enzyme inhibition also reduces superoxide production alleviating oxidative stress [32, 37]. Moreover, APO exerts a pronounced anti-inflammatory effect [38] via suppression of proinflammatory mediators and cytokines. Previous studies showed that apocynin dwindles TNF-α and IL-6 production [39]. Despite its marked activity, APO clinical utility is restricted by its low aqueous solubility, rapid elimination, and reduced bioavailability [40].

In addition to herbal drugs, many essential oils (EOs) possess anticonvulsant actions beneficial in epilepsy. Of these, Lavender oil (LAV) with its main constituent linalool were shown to inhibit CaV3.2 T-Type calcium channels by decreasing intracellular calcium thus alleviating cellular excitability and conferring protection against calcium toxicity during epileptic seizures [41, 42]. Also, LAV demonstrated nitric oxide suppressing effect in pentylenetetrazol (PTZ) kindling mouse model [43]. Previous studies proved that LAV reduced the expression of TNF-α illustrating its anti-inflammatory action [44].

In the current study, the protective antiepileptic effect of apocynin loaded lipid nanocapsules with LAV modified oily core administered subcutaneously (SC) in PTZ-induced acute seizures animal model was investigated. LAV was incorporated as a bioactive component improving drug loading efficiency while exerting an antiepileptic action. The modified LNC were further coated with lactoferrin to target specific lactoferrin receptors in brain endothelial cells. The effect of the optimized formulation on drug bioavailability and brain accumulation was studied. Moreover, the efficacy was assessed in PTZ induced seizures rat model based on severity, oxidative stress, and inflammatory markers in addition to histopathological and immunohistochemical investigations.

## Materials and methods

### Materials

Apocynin (4′-Hydroxy-3′-methoxyacetophenone) (APO) and Pentylenetetrazol (PTZ) were purchased from Sigma-Aldrich (St. Louis, MO). Labrafac^®^ lipophile WL 1349 (caprylic-capric acid triglycerides; *European Pharmacopeia*, IVth, 2002) was obtained from Gattefossé SA (Saint-Priest, France). Kolliphor HS 15 (a mixture of free polyethylene glycol 660 and polyethylene glycol 660 hydroxystearate, *European Pharmacopeia*, IVth, 2002) was obtained from BASF (Ludwigshafen, Germany). Lipoid S100 (a soybean lecithin containing 94% of phosphatidylcholine) was obtained from Lipoïd GMBH (Ludwigshafen, Germany). Lactoferrin (LF) was obtained from Lactoferrin.co (Frankfurt, Germany). Span^®^ 80 (sorbitan monooleate) was obtained from LobaChemie for Laboratory Reagents and Fine Chemicals (Mumbai, India). Lavender oil (Lavandula angustifolia aromatic 100% pure essential oil - steam extract) was obtained from Imtenan, Egypt. HPLC grade methanol was obtained from Thermo Fisher Scientific (Waltham, MA, USA). Ultracentrifugation concentration tubes (Sartorius™ Vivaspin6™, MWCO 100,000). Malondialdehyde (MDA) and Glutathione (GSH) kits (#E-BC-K030-M) were from Elabscience (Houston, Texas, USA). Superoxide Dismutase (SOD) (#ESOD-100) was purchased from BioAssay Systems (Hayward, California, USA). Rat TNF-α (#CSB-E11987r) and IL-6 ELISA kits (#CSB-E04640r) were from CUSABIO, Texas, USA. All other reagents were of analytical grade.

### Preparation of lipid nanocapsules

#### Preparation of blank and apocynin loaded LNC

LNC were formulated according to the phase inversion method with temperature cycling, antecedently described by Heurtault et al. [45] with some modifications. Briefly, equal weights of labrafac lipophile and Kolliphor HS15 (500 mg) were mixed with deionized water in the ratio 1:3 w/w. Lipoid S100 (0.75%, w/v) as a lipophilic surfactant and 0.45% w/v NaCl were added to the mixture under magnetic stirring. The mixture was subjected to three progressive heating and cooling cycles between 85 and 65 °C at 4 °C/min. An irreversible shock was engendered by four-fold dilution with cold deionized water (0–2 °C) added at 1–3 °C from the beginning of the phase inversion zone (PIZ). This was then followed by slow magnetic stirring (400 rpm) for 5 min at room temperature. APO loaded formulations were prepared by adding the drug in the concentration range (1–4 mg/mL) to the other LNC ingredients at the beginning of the formulation process. To enhance drug loading capacity, Lipoid S100 was substituted with Span 80 (0.5 and 0.8% w/v) as previously reported [46]. This was accompanied by a decrease in the temperature cycling range to 65–45 °C.

The formulation was further modified by incorporation of lavender essential oil (LAV) into the LNC oil phase. Different weight ratios of labrafac: LAV (3:1, 2:1 and 1:2) were investigated while keeping the total oil content constant (50 mg/ml of the final dispersion volume). Formulation codes and composition are listed in Table 1.

**Table 1:** Formulation code, composition and colloidal properties of blank LNC formulations (n = 3)

	*Weight ratio of Labrafac: lavender*	*Lipoid (mg/mL)*	*Span 80 (mg/mL)*	*Size (nm)*	*PDI*
*F1*	4:0	7.5	-	41.08 ± 0.3	0.13
*F2*	3:1	7.5	-	40.77 ± 0.2	0.14
*F3*	2:1	7.5	-	38.76 ± 3.3	0.21
*F4*	1:2	7.5	-	31.11 ± 2.3	0.26
*F5*	4:0	-	5	82.17 ± 0.5	0.08
*F6*	3:1	-	5	83.91 ± 1.3	0.27
*F7*	2:1	-	5	62.84 ± 1.3	0.29
*F8*	1:2	-	5	51.54 ± 5.1	0.28
*F9*	4:0	-	8	57.13 ± 1.9	0.11
*F10*	3:1	-	8	62.37 ± 2.8	0.19
*F11*	2:1	-	8	48.53 ± 2.2	0.18
*F12*	1:2	-	8	63.75 ± 9.8	0.38

#### Preparation of lactoferrin-coated LNC

For LF coating, LNC formulations were added dropwise to LF solution in the ratio 3:1 v/v with continuous gentle magnetic stirring for 1, 2 or 3 h. Different concentrations of LF (8, 12 and 16 mg/mL dispersion) were tested.

### Physicochemical characterization of LNC

#### Colloidal properties

The Z-average particle size (PS) and polydispersity index (PDI) of LNC formulations were analyzed by dynamic light scattering (DLS) using a Malvern Zetasizer^®^ at a fixed angle (173°) at 25 °C using a 4 mW He-Ne (Zetasizer^®^ Nano ZS series DTS 1060, Malvern Instruments S.A, Worcestershire, UK). Zeta potential (ZP) was determined at 25 °C using a cell voltage of 150 V and 5 mA current. Prior to analysis, the samples were suitably diluted 1:50 v/v with filtered deionized water. Measurements were performed in triplicate (n = 3).

#### Microscopical examination

The morphological features of LNC were examined by transmission electron microscopy (TEM) (Jeol, JEM-100 CX electron microscope, Tokyo, Japan). LNC dispersions were diluted (1:9 v/v) with filtered deionized water. A drop of LNC dispersion was mounted on a carbon coated copper grid then negatively stained using a 2% w/v aqueous uranyl acetate solution for 30 s. Samples were allowed to dry under ambient conditions. Shots were then taken at $\times$ 40 K magnification.

#### Entrapment efficiency and drug payload

The entrapment efficiency (EE) of APO in APO-LNC formulations was obtained by calculating the free APO concentration in the filtrate using ultrafiltration/centrifugation technique [47]. A 3 ml sample was placed in Vivaspin^®^ 6 concentrator (MWCO = 100,000, Sartorius, USA) and centrifuged at 6000 rpm for 15 min at 4 °C (Sigma 3-30KS, Sigma Laborzentrifugen GmbH, Germany). The free drug in the filtrate was determined by UV-visible spectrophotometry at 278 nm (Cary 60 UV-visible spectrophotometer, Agilent, Santa Clara, CA, USA). Method validation regarding linearity, limit of detection (LOD), limit of quantitation (LOQ) and % recovery was done. The %EE was calculated from the difference between the initial drug concentration added and the free drug concentration in the filtrate as in Eq. 1:

1 $$EE\%\;=\frac{total\; drug\; amount-unentrapped\; drug }{total\; drug\; amount }*100$$

Measurements were done in triplicate (n = 3).

Drug payload (DL) was then calculated relative to the total dry weight of the LNC formulation (Eq. 2):

2 $$DL=\;\frac{APO\; entrapped\; (mg)}{Total\; dry\; weight\; (g)}$$

### Drug release

The in vitro release of APO from LNC was determined at 37 °C and 100 rpm in phosphate buffer saline (PBS, pH 7.4) to mimic physiological conditions. Samples (200 µL) of APO-LNC, APO-LAV/LNC, LF-APO-LNC and LF-APO-LAV/LNC dispersions equivalent to 800 µg APO were diluted to 5 ml with PBS maintaining sink conditions. At predetermined time intervals (1 – 72 h), LNC were separated using ultrafiltration/ centrifugation technique. The filtrate was then analyzed for APO concentration using the UV-visible spectrophotometry as described under entrapment efficiency. The % APO released was calculated in triplicate relative to the theoretical initial drug content (n = 3).

The release kinetics were evaluated by fitting the release profiles of LNC to different mathematical kinetic models and selecting the best fit by regression analysis of the plot. This was conducted using an Excel add-in, DDsolver, for modeling and comparing different drug release profiles.

### Storage Stability

Formulations were refrigerated for 6 months at 4 °C. PS, PDI, and ZP were assessed at monthly intervals over the study period. Changes in %EE as an indicator for drug leakage were also investigated.

### In vivo study design

#### Animals

Male Wistar rats were obtained from the animal house of Faculty of Medicine, Alexandria University. Rats were housed under standard lab settings (12-h light/dark cycle). Animals were given access to food and water ad libitum. Before beginning the experiment, rats were acclimatized to the living conditions for 7 days. Experimental protocol was approved by the Institutional Animal Care and Ethics Committee of the Faculty of Pharmacy, Alexandria University, Egypt (approval code: AU-06-2023-2/162).

#### Apocynin bioavailability and brain distribution study

##### Pharmacokinetic study

Fifteen rats were randomly divided into 3 equal groups. Animals were fasted overnight prior to the experiment with free access to water. A single 30 mg/ Kg subcutaneous dose of APO [2] as APO solution in 15% PEG 400 saline solution, APO-LAV/LNC and LF-APO-LAV/LNC was administered. Blood samples were collected from 5 rats in each group via the orbital plexus under anesthesia at prescheduled time points (5, 15, 30, 45, 60 and 90 min) in EDTA-containing tubes. Blood samples were centrifuged at 5000 rpm for 10 min. Plasma samples were stored at -80 °C pending analysis.

APO in plasma samples was quantified using a previously reported method with slight modifications [48]. An aliquot of rat plasma samples (100 µL) was vortex-mixed with 400 µL methanol (HPLC grade, Sigma Aldrich) for 1 min then centrifuged at 5000 rpm for 10 min. The obtained supernatants were filtered through 0.22 µm PTFE filters. APO in plasma was determined by HPLC-UV using a previously reported method for APO separation with some modifications [48]. Agilent Technologies-1260 infinity; Santa Clara, CA, USA system set at λmax 278 nm was used. Separation was carried out on ZORBAX Eclipse Plus C18 column (4.6 × 150 mm, 5 μm) using an isocratic eluent consisting of a mixture of methanol: water (40:60, v/v) at a flow rate of 1 ml/ min. The injection volume was 20 µl and the retention time was 5–5.5 min. APO concentration was obtained using calibration curves for peak areas of APO in spiked plasma (0.0625–8 µg/ml) obtained under similar conditions. The method was validated regarding linearity, limit of detection (LOD), limit of quantitation (LOQ) and % recovery.

Plasma concentrations versus time data were plotted. Non-compartmental pharmacokinetic data analysis using the Excel pharmacokinetic solver add-in [49] was done to obtain the pharmacokinetic parameters: total area under the curve (AUC 0-inf), peak plasma concentration (Cmax), time to reach the maximum plasma concentration (Tmax), and mean residence time (MRT). Results were expressed as mean ± SD.

##### Brain distribution study

For the brain accumulation study, 18 rats were divided into 3 groups. At 45- and 90-min intervals following SC administration of APO, APO-LAV/LNC and LF-APO-LAV/LNC, blood samples were collected from 3 rats/ group and the whole brain was excised, rinsed with ice-cold saline and immediately frozen at -80 °C for APO quantification. Brains were then weighed and homogenized in ice-cold saline (1 g/1 mL). APO was extracted using methanol followed by centrifugation at 10,000 rpm for 10 min at 4 °C [50]. The obtained supernatant was filtered through 0.22 µm PTFE filters and analyzed by a validated HPLC-UV method.

Log BB of APO as an indicator of BBB permeation was calculated as described [51] using the equation:

3 $$LogBB=Log\frac{{C}_{brain}}{{C}_{plasma}}$$

where, C_brain_ and C_plasma_ are APO concentrations in brain (µg/g) and plasma (µg/mL), respectively at 45- or 90-min following SC injection of APO solution, APO-LAV-LNC and LF-APO-LAV/LNC (n = 3).

#### In vivo efficacy in PTZ induced seizures model

##### Experimental design

Forty-two rats weighing 180–200 g were randomly divided into seven groups (6 animals each) as follows: positive control group (receiving only PTZ), APO solution in 15% PEG 400 saline solution, APO-LNC, APO-LAV/LNC, LF-APO-LNC and LF-APO-LAV/LNC. Treatments were administered by SC injection of APO solution or LNC formulations equivalent to 30 mg APO/kg [2]. A healthy group (receiving only saline) was included for comparison. Except for the healthy group, all animals received pentylenetetrazol (PTZ) intraperitoneal injection (70 mg/Kg) 1 h following drug administration to induce epileptic seizures. Animals were then monitored for 40 min for seizures scoring. At the end of the experiment animals were euthanized by cervical dislocation. Brains were then excised, washed with saline, and divided for further biochemical, histopathological and immunohistochemical analysis.

##### Assessment of seizure behaviour

Following PTZ injection, animals were monitored and seizure index recorded over 40 min based on a modified Racine scale [52] as listed in Table 2. Also, latency; time between PTZ injection and onset of seizures and duration; time interval from the onset to termination of seizures or death of the animal.

**Table 2:** Racine rating scale for seizures evaluation

**Phase**	**Behavioral expression**
0	No behavioral changes
1	Ear and facial twitching
2	Myoclonic jerks without rearing
3	Myoclonic jerks with rearing
4	Turning over onto side position, tonic-clonic seizures
5	Turning over onto back position, generalized tonic-clonic seizures

##### Biochemical analysis

1. Brain homogenization
  Samples were homogenized in ice-cold 10 mM phosphate buffer (pH 7.4) to obtain a 10 %w/v homogenate. The homogenate was centrifuged at 10,000 g for 15 min at 4 °C. The supernatant formed was separated to be used for further biochemical analysis.
2. Oxidative stress markers
  - **Estimation of lipid peroxidation**
    Brain tissue homogenate was analyzed for lipid peroxidation products using a commercial MDA kit that avails of thiobarbituric acid (TBA) reaction with MDA in an acidic medium at 95 °C for 30 min to form a colored complex as previously described [53]. Tissue homogenate (0.1 ml) was pipetted into a tube containing an equal volume of SDS solution. This was followed by the addition of 0.75 ml acetic acid, 0.75 ml of TBA and 0.3 ml of distilled water. The contents of the tubes were then vortex mixed and incubated in a boiling water bath for 1 h then cooled to room temperature. An aliquot of 0.5 ml of distilled water was added to each tube followed by the addition of 2.5 ml n-butanol and vigorous mixing then centrifugation at 2500 xg for 10 min. Absorbance of the organic layer was measured spectrophotometrically at 534 nm. The level of lipid peroxidation was calculated and expressed as nmol MDA per mL.
  - **Estimation of reduced glutathione (GSH)**
    The level of GSH in the brain homogenate was determined according to Ellman’s method [54]. A colorimetric method in which Ellman’s reagent or DTNB (5,5′-dithiobis-(2-nitrobenzoic acid)) reacts with GSH forming a yellow color detectable using UV spectrophotometer at 412 nm was used. GSH colourimetric assay kit was used according to manufacturer instructions. GSH concentration was determined from the standard curve and expressed as nmol/mg protein.
  - **Determination of antioxidant capacity**
    Superoxide dismutase (SOD) activity was investigated according to the previously mentioned protocol by Misra and Fridovich [55]. The SOD activity suppression was evaluated by epinephrine oxidation and formation of adrenochrome and superoxide radicals. Following brain tissue homogenization and centrifugation the supernatant was separated. for total SOD assay following manufacturers instructions. SOD was measured at 440 nm. The results are expressed as U/mg tissue protein.
3. Estimation of pro-inflammatory markers
  The levels of the proinflammatory cytokines; tumor necrosis factor-α (TNF-α) and interleukin-6 (IL-6) in brain tissue homogenates were measured using ELISA kits according to the manufacturer’s instructions.

##### Histopathological examination

Brain samples were excised and post-fixed for 48 h at room temperature in 4% paraformaldehyde. Specimens were fixed in 5% buffered formalin, dried, and cleaned before being embedded in paraffin. Serial sections with a five-micrometer thickness were placed on slides coated with poly-L-lysine, deparaffinized in xylene, and rehydrated in ethanol. The serial sections were then stained with hematoxylin and eosin (H&E) in accordance with the standard procedure for histological analysis of sections from various groups [56, 57]. Representative photographs were captured at different magnifications of each experimental group with an Olympus UC30 digital camera and an Olympus XC30 microscope (Germany).

##### Immunohistochemical analysis

Epitope retrieval was accomplished before immunohistochemistry by microwaving slide mounted specimens in a 10 mM sodium citrate (pH 6.0) solution for three minutes to enhance staining. Brain sections were incubated for 15 min in a 5% bovine serum albumin solution in PBS with 0.1% Triton X-100 after washing with phosphate buffer solution (PBS, 0.25 M, pH 7.4). Additionally, samples were treated with primary antibodies for detection of glial fibrillary acidic protein (GFAP) in astrocytes and caspase-3 [58]. For quantitative analysis, two independent observers manually counted the numbers of positive cells in five randomly chosen fields for each antibody using the image processing tool Image J.

### Statistical analysis

All experiments were conducted in triplicate (n = 3), and results were expressed as mean ± SD. Statistical analyses were implemented using an unpaired Student’s t-test and one-way analysis of variance (ANOVA) followed by a post-hoc Tukey’s test for multiple comparisons using GraphPad Prism (Version 7.04, San Diego, CA, USA). Significant difference was set at p ≤ 0.05.

## Results and discussion

### Preparation of LNC

As patented by Heurtault et al. [45], the basic composition of LNC formulations includes the medium chain triglyceride; Labrafac as the oily core with the pegylated surfactant; Kolliphor HS15 and Lipoid S100 as the outer tensioactive shell. In the current study, the basic formula (F1) containing equal weights of labrafac lipophile and Kolliphor HS15 (500 mg) in the ratio 1:3 w/w to deionized water was selected based on previous reports [20, 59]. The oily core was modified by inclusion of lavender essential oil (LAV) due to its well reported anticonvulsant effect [60]. LAV was incorporated into the LNC formulation in different Labrafac: LAV ratios (3:1, 2:1 and 1:2). Inclusion of LAV in the formulation did not affect its clarity. Moreover, to increase APO loading, the composition was further modified by replacing Lipoid S100 with Span 80 as reported earlier [46]. Unlike Lipoid S100 entailing heating/cooling cycles in the range 65–85 °C, incorporation of span 80 necessitated a lower temperature range (45–65 °C) [61, 62]. This was previously attributed to its ability to reduce oil and water interfacial tension thus increasing interface fluidity [61].

### Colloidal properties

Colloidal properties of LNC were previously shown to be dependent on composition (Oil: SAA ratio) with particle size ranging from 25 to 100 nm [63–65]. Table 1 shows the PS, PDI and ZP of LNC. Blank LNC prepared with Lipoid S100 at different Labrafac: LAV ratios (F1-F4) showed good colloidal properties with relatively small particle size (31.1 - 41.1 nm) and narrow size distribution (PDI ≤ 0.3). This reflects the well reported monodispersity of LNC [45, 63]. Increasing the proportion of LAV in LNC resulted in a decrease in PS with an increase in PDI but the difference was only statistically significant (p ≤ 0.05) when the proportion of LAV exceeded that of Labrafac (1:2; Labrafac: LAV ratio, F4). El-Tokhy et al.,[65] previously reported smaller PS for LNC with Lavander essential oil oily core compared to Labrafac. This was attributed to Lavender oil low viscosity and low oil/water interfacial tension in addition to its polarity and hence ability to form nanoemulsions by the phase-inversion method [65].

Following replacement of Lipoid S100 with span 80, a significant increase in particle size (p ≤ 0.05) was observed. The obtained PS is in agreement with previous reports on Span 80 modified LNC where the PS observed was in the range of ⁓55–70 nm [46, 62]. Two different concentrations of span 80 (5 and 8 mg/mL) were tested. Increasing the concentration of span 80 while keeping the labrafac: LAV ratio brought about a significant decrease in particle size (p ≤ 0.05) for all Labrafac: LAV ratios tested (4:0, 3:1 and 2:1) except for the ratio 1:2 where an insignificant change in PS accompanied the increase in span 80 (p > 0.05). Moreover, with increasing span 80 from 5 to 8 mg/mL, a significant decrease in PDI was observed for LAV containing formulations except for F12 with Labrafac: LAV ratio 1:2. It is worth noting that all span 80 containing formulations showed a PDI below 0.3 indicating monodispersity except for F12 which showed a PDI of 0.38.

Regarding ZP, an average negative charge of -9.1 ± 0.8 mV was observed for lipoid containing formulations which was not affected by LAV incorporation. The negative zeta potential is probably due to the presence of a small proportion of hydrolyzed surfactants [66] together with the formation of Kolliphor HS15 intramolecular hydrogen bonding [67]. A slight increase in ZP was observed following Span 80 incorporation (-11.1 ± 0.5 mV). This could be attributed to the presence of a small fraction of free fatty acids in the co-surfactant, leading to the presentation of negatively charged polar groups [68].

In all formulations APO loading resulted in an insignificant change in colloidal properties (p > 0.05).

### UV-spectrophotometric method validation

APO was analyzed spectrophotometrically at 278 nm. Linearity was investigated in the range 2–10 µg/mL with a coefficient of determination (R^2^) 0.99. The LOD and LOQ were 0.643 and 1.95 µg/mL, respectively. Recovery percentage ranged from 99% to 100.25%. The intra-day and inter-day precision were 3.29% and 3.41%, respectively.

### Entrapment efficiency and drug payload

For %EE determination, APO was analyzed using the validated UV spectrophotometric method at 278 nm.

Lipoid S100 containing formulations showed an average %EE of 79.5 ± 2.8% with a drug payload ⁓11 mg/g for F1-F4 formulations following loading with 2 mg/mL APO. Above this concentration, APO precipitation was evident. Replacement of lipoid with 5 mg/mL Span 80 allowed for incorporation of up to 4 mg/mL APO with an average %EE of 82.1 ± 1.9% and a drug payload of ⁓23 mg/g for F5-F8 formulations. Further increase in span 80 to 8 mg/mL (F9-F12) allowed for a significantly higher %EE (p ≤ 0.05) reaching an average of 87.4 ± 2.4% and a drug payload ⁓25 mg/g.

These observations reflect the dual benefit of increasing span 80 concentration from 5 to 8 mg/mL; it reduced particle size with a narrower size distribution and was essential to boost drug loading efficiency.

As evident from the low SD values, varying the proportion of LAV did not significantly affect APO %EE (p > 0.05). It is worth mentioning that LNC allowed for higher APO encapsulation compared to the previously prepared solid lipid nanoparticles that showed a maximum EE% of 30.5 ± 2.25% [40].

Based on the characterization of colloidal properties and drug loading efficiency, F11 was selected as the optimum LAV modified formulation with maximum APO loading (APO-LAV/LNC) for LF coating and further characterization.

### Lactoferrin coating

LF coating was applied to the selected optimized LNC formulations (F9 and F11) as a specific brain targeting ligand, augmenting BBB penetration and brain delivery of nanoparticles [69]. LF is a pervasively investigated natural cationic protein with ease of application as a coating layer on negatively charged nanoparticles by electrostatic interaction [70]. The positively charged nature of LF facilitates its uptake by anionic cell membranes. In addition, it possesses the ability to actively target damaged brain cells by specifically binding to lactoferrin and transferrin receptors [71]. Therefore, LF coating of LNC is expected to enhance the anticonvulsant effect. Efficient coating of LNC was judged through changes in PS and ZP [72]. Owing to the electrostatic deposition of a cationic LF layer on the anionic LNC, a decrease in the negativity of the LF-coated formulae with a slight increase in PS indicates successful coating [73].

**Fig. 1:**
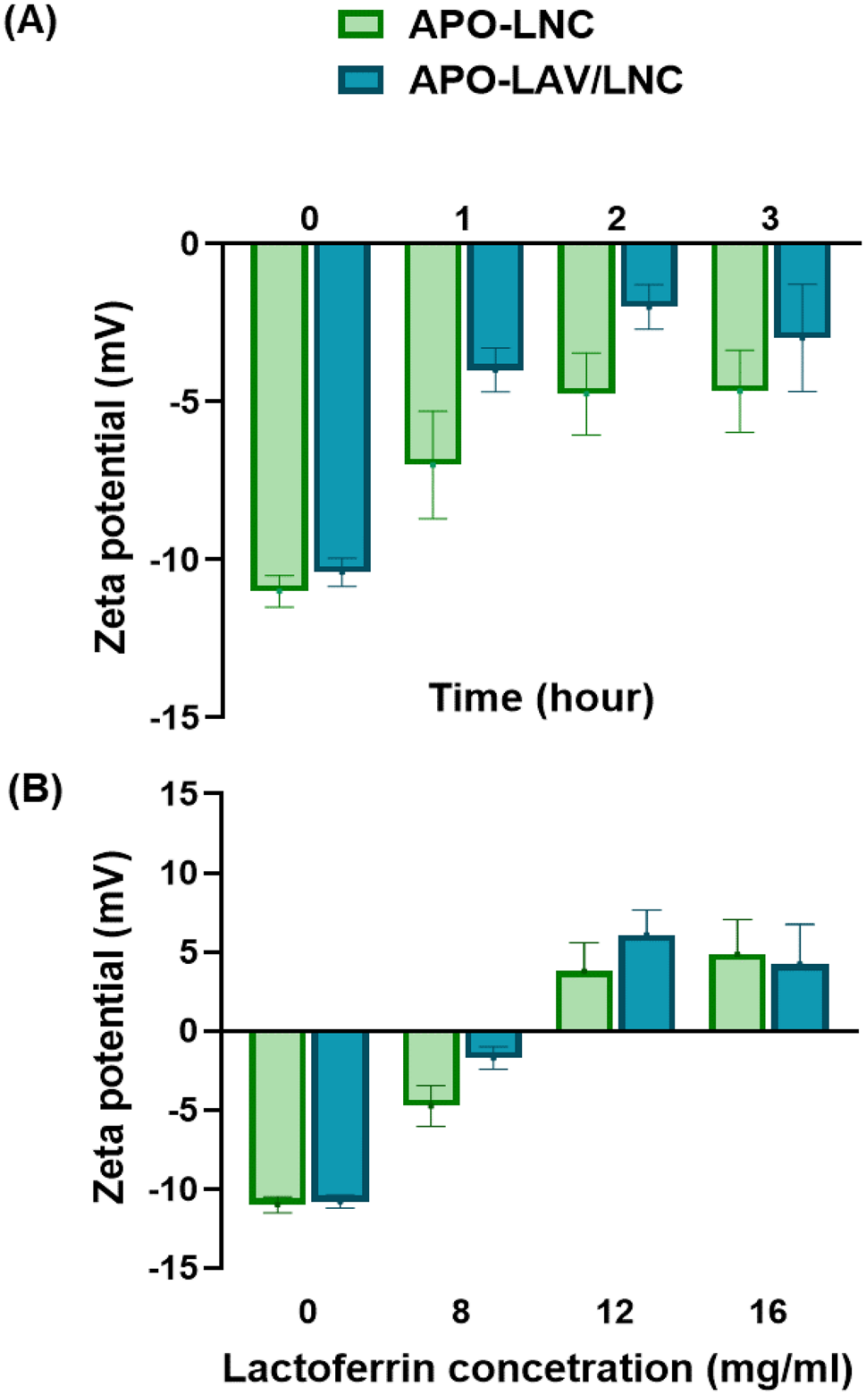
Optimization of lactoferrin coated APO-LNC and APO-LAV/LNC in terms of **A** stirring time, h and **B** lactoferrin concentration, mg/mL

First, stirring period following addition of the formulation to LF solution at a concentration 8 mg/mL was optimized. This was done for 1, 2 and 3 h (Fig. 1A). The results showed a significant change in ZP (p ≤ 0.05) by increasing stirring duration from 1 to 2 h. A further increase in time did not result in a significant difference in ZP (p > 0.05) and hence, 2 h was selected as the optimum stirring duration.

To select the optimum LF concentration, coating with 8, 12 and 16 mg/mL was tested (Fig. 1B). At 8 mg/mL concentration, a significant reduction in negative charge was observed for both APO-LNC and APO-LAV/LNC (-4.75 ± 1.3 and -2 ± 0.7 mV, respectively). An increase in LF concentration to 12 mg/mL brought about a shift in ZP from negative to positive charge (3.77 ± 1.8 and 6.07 ± 1.6 mV for APO-LNC and APO-LAV/LNC, respectively). This was not significantly changed following an increase to 16 mg/mL (p > 0.05). Therefore, 12 mg/mL was selected as the optimum LF concentration. The significant increase in PS (p ≤ 0.05) at this concentration is also confirmatory of successful coating. Herein, APO-LNC showed an increase in PS from 57.1 ± 1.9 nm to 74.1 ± 4.1 nm for LF-APO-LNC (p ≤ 0.05). Also, LF-APO-LAV/LNC showed a significantly higher PS (p ≤ 0.05) compared to APO-LAV/LNC (59.7 ± 4.5 and 48.5 ± 2.2 nm, respectively).

Moreover, %EE was not significantly affected by LF coating (p > 0.05) where LF coated formulations showed a %EE of 88 ± 1.75% and 92 ± 2.4% for LF-APO-LNC and LF-APO-LAV/LNC, respectively.

### Microscopical examination

**Fig. 2:**
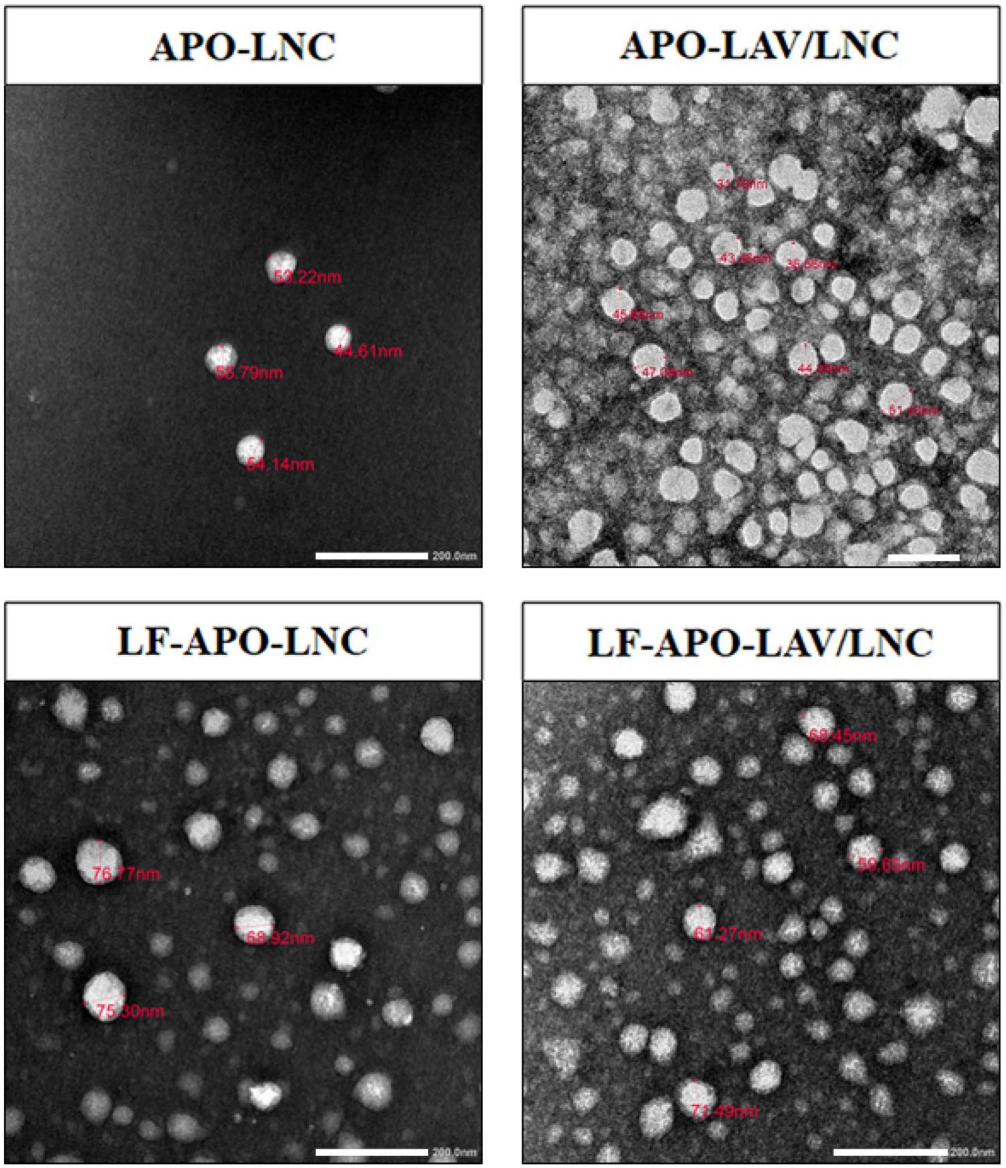
TEM images of APO-LNC, APO-LAV/LNC, LF-APO-LNC and LF-APO-LAV/LNC × 40,000. The scale bar represents 200 nm

TEM examination of the selected APO loaded LNC formulations revealed nearly spherical and homogenously distributed nanostructures without aggregation (Fig. 2). The perceived morphological features concur with those previously reported for LNC [15, 66]. The observations are also in accordance with those obtained by DLS showing an increase in size following LF coating for both APO-LNC and APO-LAV/LNC.

### In vitro drug release

**Fig. 3:**
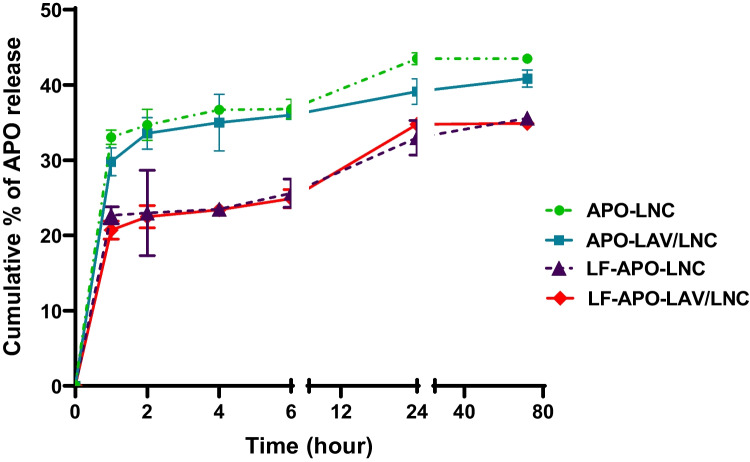
Apocynin release profile from LNC formulations over 72 h at 37 °C (n = 3; data are shown as mean ± SD)

The in vitro release profile of APO-LNC and APO-LAV/LNC and their LF coated counterparts over 72 h is shown in (Fig. 3). APO release exhibited a biphasic release profile with an initial burst during the first hour followed by sustained release reaching a maximum of ⁓44% over 72 h for APO-LNC. LAV modified formulation (APO-LAV/LNC) showed slight insignificant reduction in drug release with ⁓ 41% released after 72 h (p > 0.05). The highly sustained release rate ensures successful drug encapsulation in the oily core. This is in accordance with earlier reports on lipophilic drug release patterns from LNC formulations [20, 47, 59]. The release of lipophilic drugs from LNC comprises firstly diffusion of the solubilized drug in the oily core followed by partitioning into aqueous surrounding release medium presenting a barrier that hampers drug transport to the surrounding aqueous medium [62].

For LF coated formulations significantly reduced release rates reaching ⁓ 36 and 35% at 72 h interval for LF-APO-LNC and LF-APO-LAV/LNC, respectively (p ≤ 0.05) were observed. The reduced APO release from LF coated LNC could be attributed to the steric hindrance conferred by the LF coat which acts as an additional diffusional barrier controlling drug release [74].

The mechanism of drug release from APO-LNC, APO-LAV-LNC, LF-APO- LNC and LF-APO-LAV/LNC was assessed by fitting to different release kinetics models; zero-order, first-order, Higuchi, Korsmeyer–Peppas, and Hixson–Crowell models. The statistical parameters used to nominate data best fit were the highest correlation coefficient (r) and the lowest mean standard error (MSE) (Table 3). For the four formulations examined, Korsmeyer–Peppas showed the greatest r value (0.99) and the lowest MSE indicating that APO was released by diffusion-controlled release. Also, the release exponent value (n) was below 0.5 indicative of Fickian diffusion.

**Table 3:** Kinetics modeling of drug release from different LNC formulations

**Formula**	**APO-LNC**		**APO-LAV/LNC**		**LF-APO-LNC**		**LF-APO-LAV/LNC**	
	MSE	r	MSE	r	MSE	r	MSE	r
**Zero-order**	844.2	0.465	751.6	0.453	384.6	0.621	372.3	0.625
**First-order**	738.1	0.646	672.1	0.494	346.8	0.652	333.3	0.662
**Higuchi**	414.9	0.646	374	0.632	165.3	0.778	154.2	0.791
**Hixon-crowell**	780.1	0.496	702.8	0.477	360.6	0.641	347.5	0.648
**Korsmeyer-peppas**	1.33	0.997	0.95	0.998	1.68	0.995	3.28	0.993
	n = 0.07		n = 0.066		n = 0.123		n = 0.137	

*MSE* mean standard error, *r* correlation coefficient

### Storage stability

**Fig. 4:**
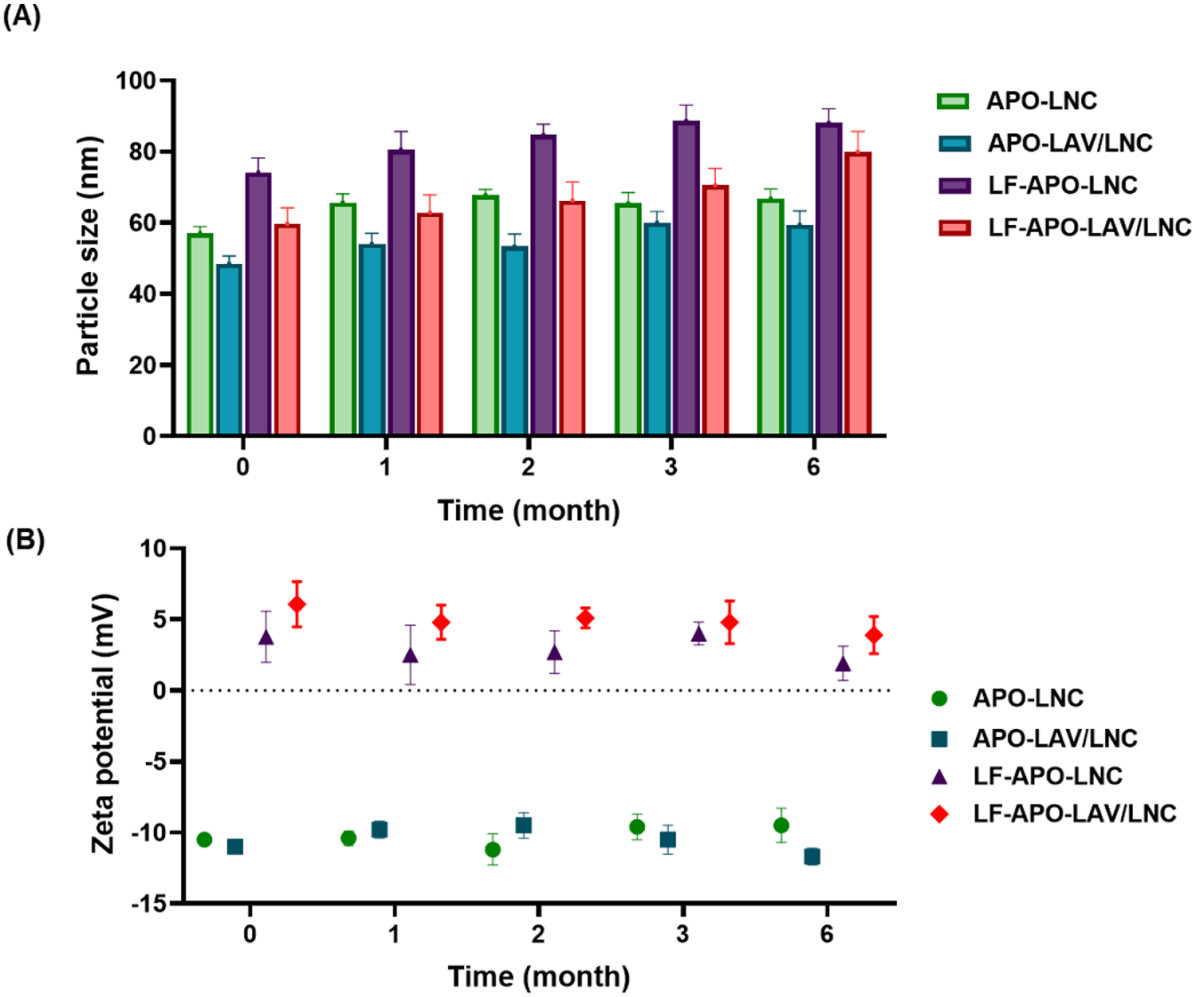
Change in **A** particle size, and **B** zeta potential of APO LNC formulations upon storage at 4 °C over 6 months (n = 3)

The stability of the selected formulations was studied over 6 months at 4 °C (Fig. 4). This was judged by monitoring changes in colloidal properties and drug %EE. Regarding PS (Fig. 4A), APO-LNC showed a slight increase in PS after 1 month (from 57.1 ± 1.9 to 65.7 ± 2.5 nm) which was stable thereafter (66.8 ± 2.7 nm at the 6 months interval). On the other hand, APO-LAV/LNC did not show any significant change in PS over 2 months (p > 0.05) with only a slight increase in PS at the three-month interval (59.9 ± 4.5 nm) which again did not change at the 6-month interval (59.4 ± 3.9 nm). This colloidal stability could be a result of the steric hindrance exerted by the bulky PEG chains of Kolliphor^®^ HS 15 [67]. Also, the LF coated formulations showed a slight change in PS over the study period which reached statistical significance only at the 6 months interval (p ≤ 0.05). The colloidal stability of the LF coated formulations despite their slight positive charge is probably conferred by the steric hindrance offered by both the bulky PEG chains of Kolliphor^®^ HS 15 and the LF coating layer. Tian et al. previously attributed the stability of the nano system and absence of aggregates to the steric hindrance of the coating layer [75]. Also, the PDI was always below 0.3 indicating monodispersity of the system.

The effect of storage on the ZP of the test formulations was studied (Fig. 4B). The results showed no significant changes in ZP throughout the study. For the LF coated formulations the slight changes in ZP observed reflect the stability of the coat over time.

As a measure of drug escape from the system upon storage, %EE was reassessed at each time point. %EE remained above 85% with no significant decrease in drug entrapment all through the storage period (p > 0.05) for any of the formulations tested.

### Pharmacokinetic profile and brain distribution study

#### HPLC method validation

Linearity was checked in the concentration range 0.0625-08 µg/mL showing a determination coefficient 0.999. The limits of detection (LOD) and quantitation (LOQ) were 0.063 and 0.192 µg/mL, respectively. Recovery percentage ranged from 94- 98.89%.

#### Pharmacokinetics

**Fig. 5:**
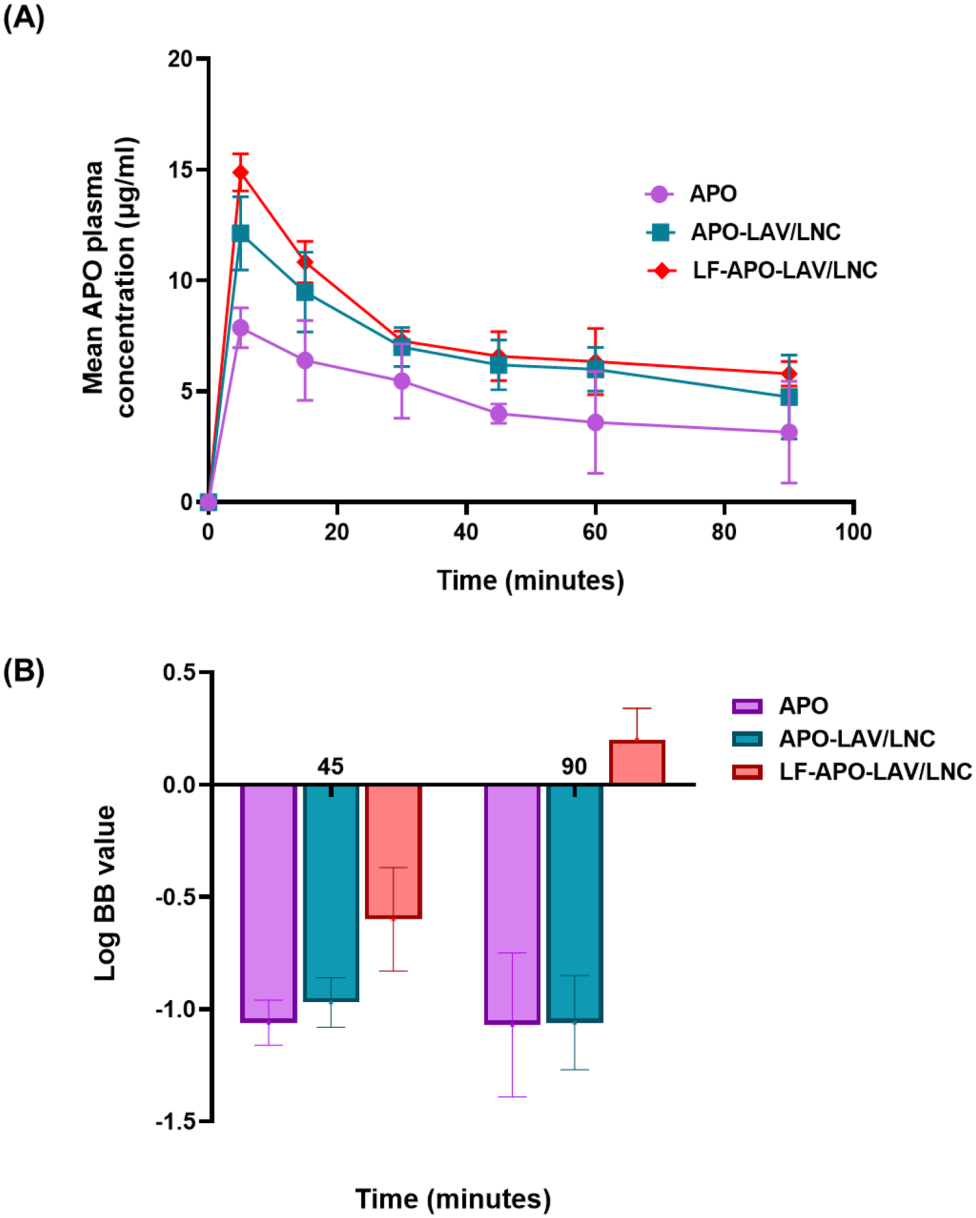
**A **Mean plasma concentration – time profile of APO following subcutaneous administration of a single 30 mg/Kg dose of APO solution or APO-LNC formulations to rats (n = 5) and **B** LogBB values as an indicator of BBB permeability at 45 and 90 min intervals (n = 3). Data presented as mean ± standard deviation (SD)

The bioavailability of APO administered via SC injection was investigated for free APO, APO-LAV/LNC, LF-APO-LAV/LNC. The drug-plasma concentration-time profile after SC injection of a single APO dose (30mg/kg) either free or loaded into LNC formulations is presented in Fig. 5A and the pharmacokinetic parameters are listed in Table 4. The results showed obvious changes in the pharmacokinetic behavior of APO after loading into both APO-LAV/LNC and LF-APO-LAV/LNC. APO was detected in blood 5 min after injection confirming its rapid absorption from the injection site to the blood circulation. Whereas T_max_ was not affected by APO loading into LNC formulations, a significant change in C_max_ was observed (1.5- and 1.9-fold increase for APO-LAV/LNC and LF-APO-LAV/LNC, respectively compared to APO, p ≤ 0.05) with an insignificant difference between the 2 formulations (p > 0.05). Also, a notable increase in AUC_0-t_ (p > 0.05) compared to APO was observed following encapsulation into APO-LAV/LNC and LF-APO-LAV/LNC. Despite the increase in C_max_ and AUC_0-t_ achieved by Apo-LAV/LNC, no significant increase in MRT was observed (p > 0.05). On the contrary, LF-APO-LAV/LNC brought about a 1.8-fold increase in MRT compared to APO.

**Table 4:** Pharmacokinetic parameters following subcutaneous administration of (30 mg/Kg) APO or APO-LNC formulations in rats (n = 5)

**Parameter**	**APO solution**	**APO-LAV/LNC**	**LF-APO-LAV/LNC**
**T**_**max**_** (min)**	5	5	5
**C**_**max**_** (µg/mL)**	7.87 ± 0.98	12.13 ± 1.8	14.88 ± 2.7
**AUC**_**0-t**_ (µg/mL*min)	402.39 ± 16.4	613.725 ± 25.6	685.025 ± 23.4
**AUC**_**0-∞**_ (µg/mL*min)	1018.71 ± 97	1374.02 ± 158	2710.37 ± 187
**MRT (min)**	187.79 ± 15	155.899 ± 16	338.05 ± 9.8

#### LogBB calculation

LogBB ratio is defined as the brain: blood ratio at predefined time points after drug administration to measure the transport of small drug molecules across the BBB [76]. Drugs possessing similar BBB permeability may differ in LogBB value due to differential drug binding to brain tissue [77]. LogBB is dependent on drug permeability through the BBB to reach the central nervous system [51]. If LogBB exceeds 0.3 it indicates the drug readily crossed the BBB and entered the CNS; on the other hand, if the value is below -1, the drug is poorly distributed to the brain [78, 79]. Intermediate values reflect the ability to cross the BBB [78]. Liu et al. previously reported the ability of APO to cross the BBB following IV bolus with a Log BB value − 0.1 (at 1 min) and 1 (at 30 min) [79]. In the current study, APO showed a LogBB value ⁓ (-1) at 45 min which further decreased after 90 min indicating drug clearance from the brain. APO loading into LNC did not significantly affect its brain permeability. Interestingly, LF-APO-LAV/LNC showed the highest LogBB values of -0.6 ± 0.23 and 0.2 ± 0.14 at 45 and 90 min, respectively post SC injection indicating that drug permeability across BBB and brain accumulation increased by time following LF coating. Also, it was previously proved by Kaili Hu et al. [80], that LF coating of PLGA nanoparticles facilitated access of the drug to the brain.

## In vivo study

### Effect of APO-LNC on PTZ-induced seizures

In the current study, seizures were induced by the gamma-butyric acid antagonist, PTZ which is known to instigate seizures by interacting with the benzodiazepine recognition sites in GABA_A_ receptors [81] resulting in suppression of the opening of chloride channels and alteration in the level of neurotransmitters. On the cellular level, PTZ affects various pathways leading to brain cells excitability. From these pathways is the oxidative state where it perturbs the brain antioxidant capability leading to a decrease in total glutathione [82]. It also exerts a major role in eliciting inflammatory markers such as TNF-α, IL-6 and COX II [83]. A PTZ injection (75mg/kg) was used to successfully induce seizures in rats [56]. Seizures were observed as tonic, myoclonic, and general seizures. Seizure assessment was done in terms of latency, duration and Racine scale (Fig. 5).

**Fig. 6:**
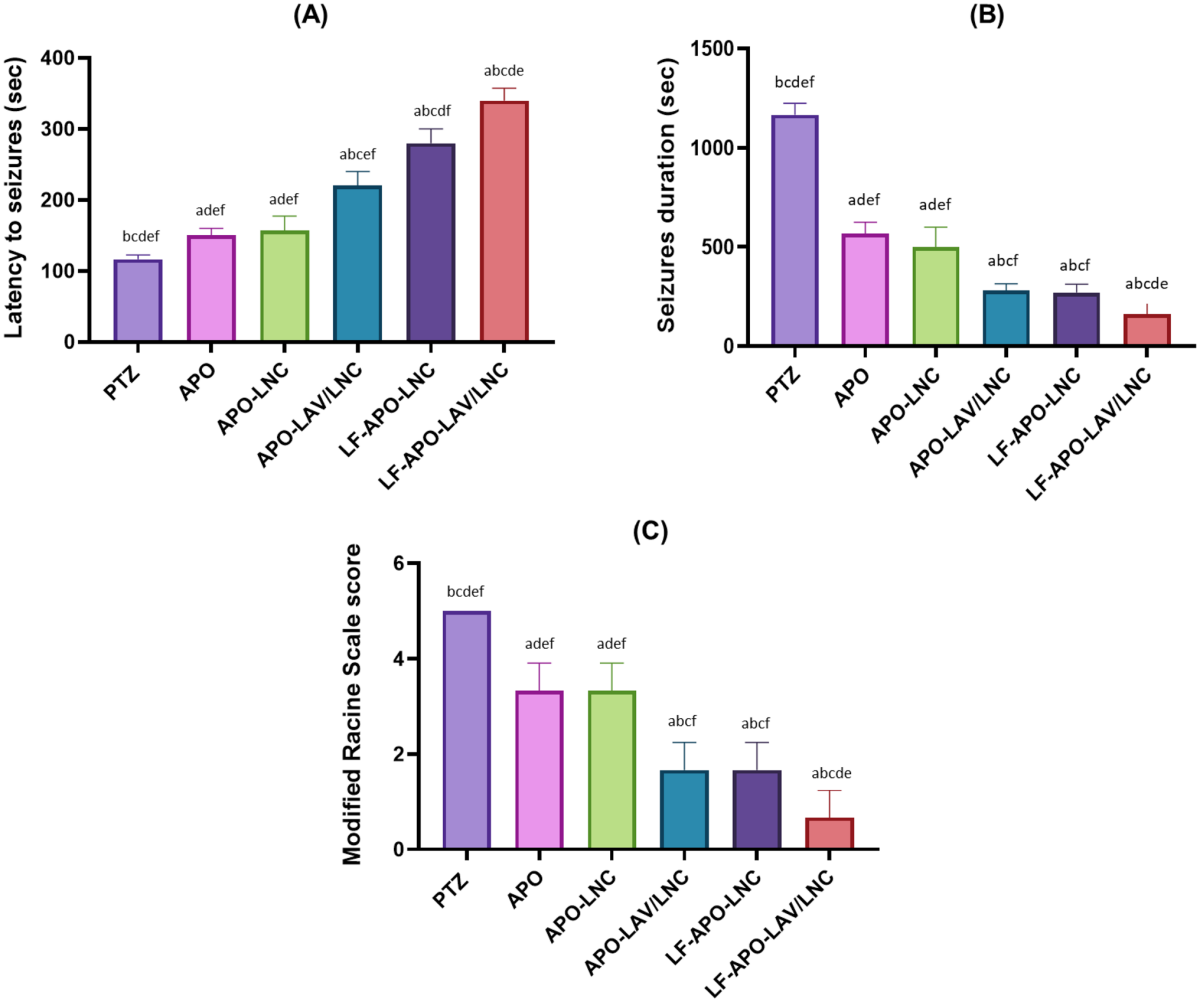
Effects of subcutaneously administered APO, APO-LNC, APO-LAV/LNC, LF-APO-LNC, LF-APO-LAV/LNC on **A** latency, **B** seizures duration and **C** behavioral changes expressed as Racine scale following PTZ- induced seizures. Data presented as mean ± standard deviation (SD) (n = 6). (^a^p ≤ 0.05 vs healthy control, ^b^p ≤ 0.05 vs positive control (PTZ), ^c^p ≤ 0.05 vs APO, ^d^p ≤ 0.05 vs APO-LNC, ^e^p ≤ 0.05 vs APO-LAV/LNC, ^f^p ≤ 0.05 vs LF-APO-LNC and ^g^p ≤ 0.05 vs LF-APO-LAV/LNC)

Rats displaying a score 3 to 5 for at least 5 min reflect development of status epilepticus [84]. In PTZ group (positive control) prominent and prolonged epileptic seizures lasting for 20 min with a mean seizure latency of 2 min and a Racine scale score of 5 were seen (Fig. 6A–C). The protective antiepileptic action of subcutaneously injected APO loaded LNC formulations (APO-LNC, APO-LAV/LNC, LF-APO-LNC and LF-APO-LAV/LNC) compared to APO solution (30 mg/kg) was done. Pre-administration of APO and APO-LNC formulation 1h before PTZ injection delayed the onset and decreased the duration of convulsions compared to PTZ group reflecting the anticonvulsant and neuroprotective efficacy of APO [2] which will be further investigated biochemically and histopathologically. APO antiepileptic effect was more pronounced following formulation modification with LAV (APO-LAV/LNC) and LF (LF-APO-LNC). LF-APO-LAV/LNC combining the merits of APO, LAV and LF on controlling epileptic seizures showed a 6 min latency and only 2 min seizure duration with a Racine scale score of 0.67 ± 0.47 and a 100% survival rate.

#### Biochemical analysis

##### Determination of oxidative stress markers

**Fig. 7:**
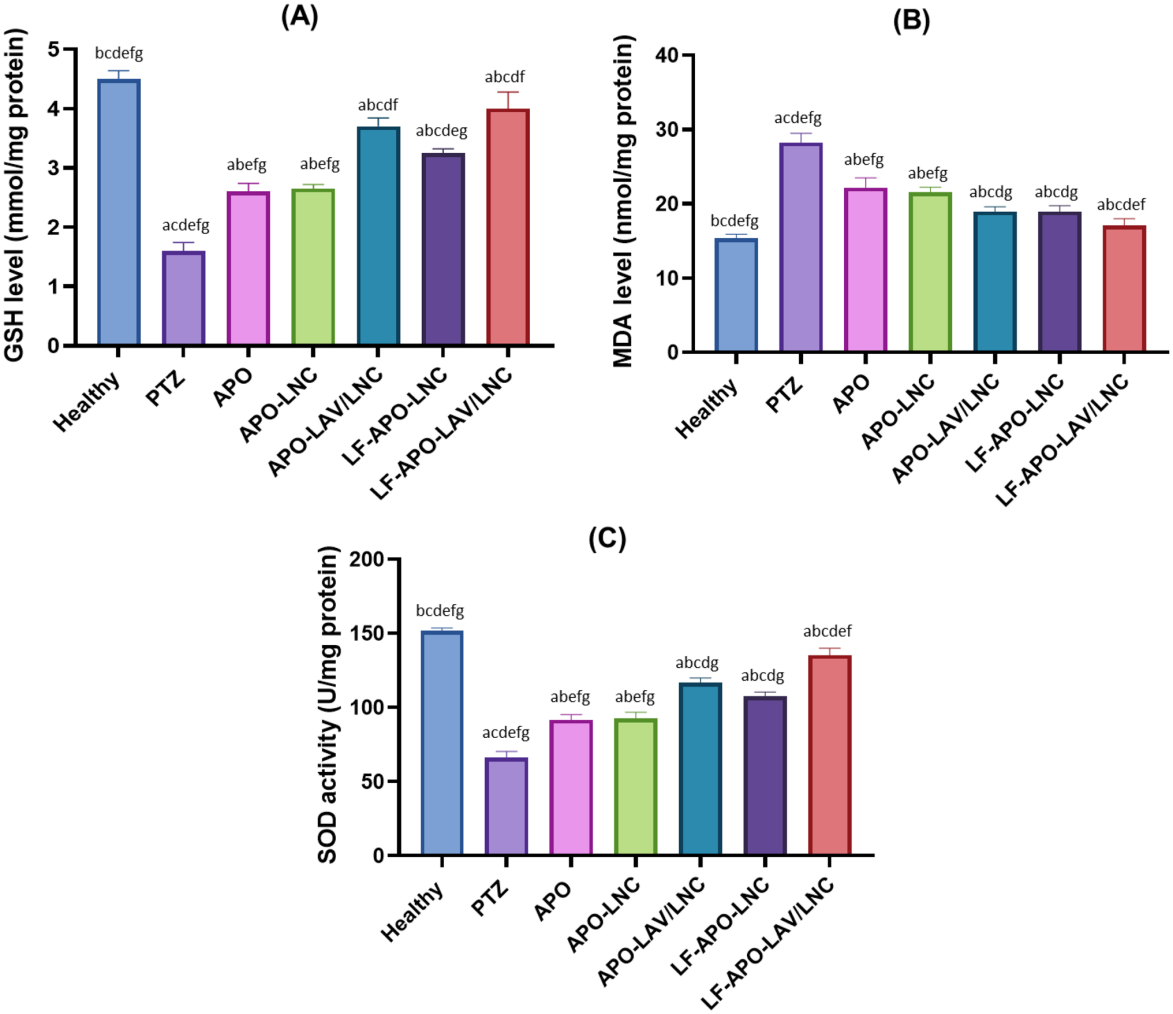
Effect of APO and APO loaded LNC formulations on pretreatment oxidative indicis: **A** reduced glutathione (GSH), **B** malondialdehyde (MDA) and **C** antioxidant capacity indicated by superoxide dismutase (SOD) level in brain tissue homogenate in a PTZ-induced seizures model in rats. Data presented as mean ± standard deviation (SD) (n = 6). (^a^p ≤ 0.05 vs healthy control, ^b^p ≤ 0.05 vs positive control (PTZ), ^c^p ≤ 0.05 vs APO, ^d^p ≤ 0.05 vs APO-LNC, ^e^p ≤ 0.05 vs APO-LAV/LNC, ^f^p ≤ 0.05 vs LF-APO-LNC and ^g^p ≤ 0.05 vs LF-APO-LAV/LNC)

Brain tissue is characterized by high unsaturated fatty acids constituent, high oxygen demand and low antioxidant capacity making it especially assailable to attack by free radicals and oxidative damage [85]. Oxidative stress has been reported as an important element in the inception and progression of epileptic seizures in several animal models including PTZ [82]. Reactive oxygen species (ROS) generation in brain tissue following epileptic insult can cause direct damage of lipids, nucleic acids, and proteins [7]. Reduced glutathione (GSH) is one of the main endogenous antioxidants in the brain with significant protective effect against lipid peroxidation (LPO). LPO is an indicator of permanent biological damage to the cell membrane phospholipid where unsaturated fatty acids in the neuronal membrane interact with hydroxyl radicals in the lipid peroxidation cascade leading to the formation of MDA, hydroxide radicals and lipid peroxide [86]. In addition to this non-enzymatic antioxidant defense mechanism, ROS are scavenged by antioxidant enzymes such as superoxide dismutase (SOD) [56]. Hence, antioxidant treatment is expected to provide neuroprotective effect via reducing oxidative stress in epilepsy [87]. In this context, the effect of pretreatment with APO and its loaded LNC formulations on the levels of GSH, MDA and SOD were examined (Fig. 7A–C).

- Reduced glutathione
  Epileptic seizures in human and animal models are accompanied by a reduction in GSH in brain tissue due to exaggerated production of reactive oxygen species [82]. This reduction increases with repetitive seizures [56, 88]. In this work (Fig. 7A), PTZ resulted in a threefold decline in GSH compared to healthy rats. Treatment with APO or APO-LNC formulations resulted in a significant increase in GSH in brain tissue homogenate compared to the PTZ group in the order LF-APO-LAV/LNC > APO-LAV/LNC > LF-APO-LNC > APO-LNC > APO (p ≤ 0.05). Both APO and APO-LNC brought about a 1.6- fold increase in GSH with no significant difference between the two groups (p > 0.05). Modifying LNC with LAV (APO-LAV/LNC) or coating with LF (LF-APO-LAV/LNC) resulted in significant enhancement in the effect of APO-LNC (p ≤ 0.05). Interestingly, the formulation combining both LAV and LF coating (LF-APO-LAV/LNC) succeeded in raising GSH to a level comparable to the healthy group (4.1 ± 0.2 and 4.6 ± 0.1 mmol/g protein, respectively).
- Lipid peroxidation
  Samples were homogenized in ice-cold 10 mM phosphate buffer (pH 7.4) to obtain a 10 %w/v homogenate. The homogenate was centrifuged at 10,000 g for 15 min at 4 °C. The supernatant formed was separated to be used for further biochemical analysis.
  The escalation of MDA values reflects tissue damage with hydroxyl radicals on the unsaturated fatty acids and collapse of antioxidant defense mechanism [89]. MDA is the end product in the oxidative cascade in damaged tissue reflecting the level of oxidative damage. Figure 7B shows a 2-fold increase in MDA in brain homogenate following PTZ administration compared to the healthy group. Pretreatment with APO-solution and APO-LNC reduced MDA level in epileptic rats (22 and 24% reduction compared to the PTZ group, respectively) again with no significant difference between the two groups (p > 0.05). Modifying LNC with the bioactive oil LAV (APO-LAV/LNC) resulted in 36 % reduction compared to PTZ group. Post insertion of LF (LF-APO-LNC and LF-APO-LAV/LNC) brought about further significant decrease in MDA level (p ≤ 0.05) compared to their uncoated counterparts (31 and 40% reduction compared to PTZ group, respectively).
- Superoxide dismutase
  Oxidative stress provoked by PTZ injection is associated with reduced levels of SOD compared to healthy group. This depletion in SOD activity is accompanied by epileptic seizures due to excessive ROS production. As presented in Fig. 7C, the positive control (PTZ group) revealed a statistically significant (p ≤ 0.05) drop in SOD level (⁓60%) when compared to the healthy group. This could be explained by the overproduction of free radicals and depletion of antioxidants in free radicals scavenging process [7]. Again, a similar pattern of antioxidant activity was observed following pretreatment with APO and APO loaded LNC formulations (Fig. 7C) with LF-APO-LAV/LNC exhibiting the maximum detoxifying role via escalation of SOD significantly relative to PTZ, APO and other APO-LNC formulations (p ≤ 0.05) with an SOD value comparable to the healthy group.
  Overall, the assessment of oxidant/antioxidant capacity of APO supports previous reports on its neuroprotective role in PTZ induced seizures [2] and ability to efficiently inhibit ROS in the brain [79] by NADPH-oxidase enzyme inhibition and diminishing superoxide production. The insignificant difference between APO and APO-LNC mirrors APO ability to cross the blood brain barrier [79] which was not improved by LNC encapsulation. The synergistic effect observed following LNC modification with LAV or LF reflects the lipid peroxidation alleviating effects of LAV [43] and LF [90]. Also, the potentiated effect of the LF coated formulations compared to the uncoated ones could result from the ability of LF to target brain tissues enhancing BBB permeability via lactoferrin-mediated transcytosis [90]. Also, the potentiated effect of the LF coated formulations compared to the uncoated ones could result from the ability of LF to target brain tissues enhancing BBB permeability via lactoferrin-mediated transcytosis [91].

##### Anti-inflammatory effect

**Fig. 8:**
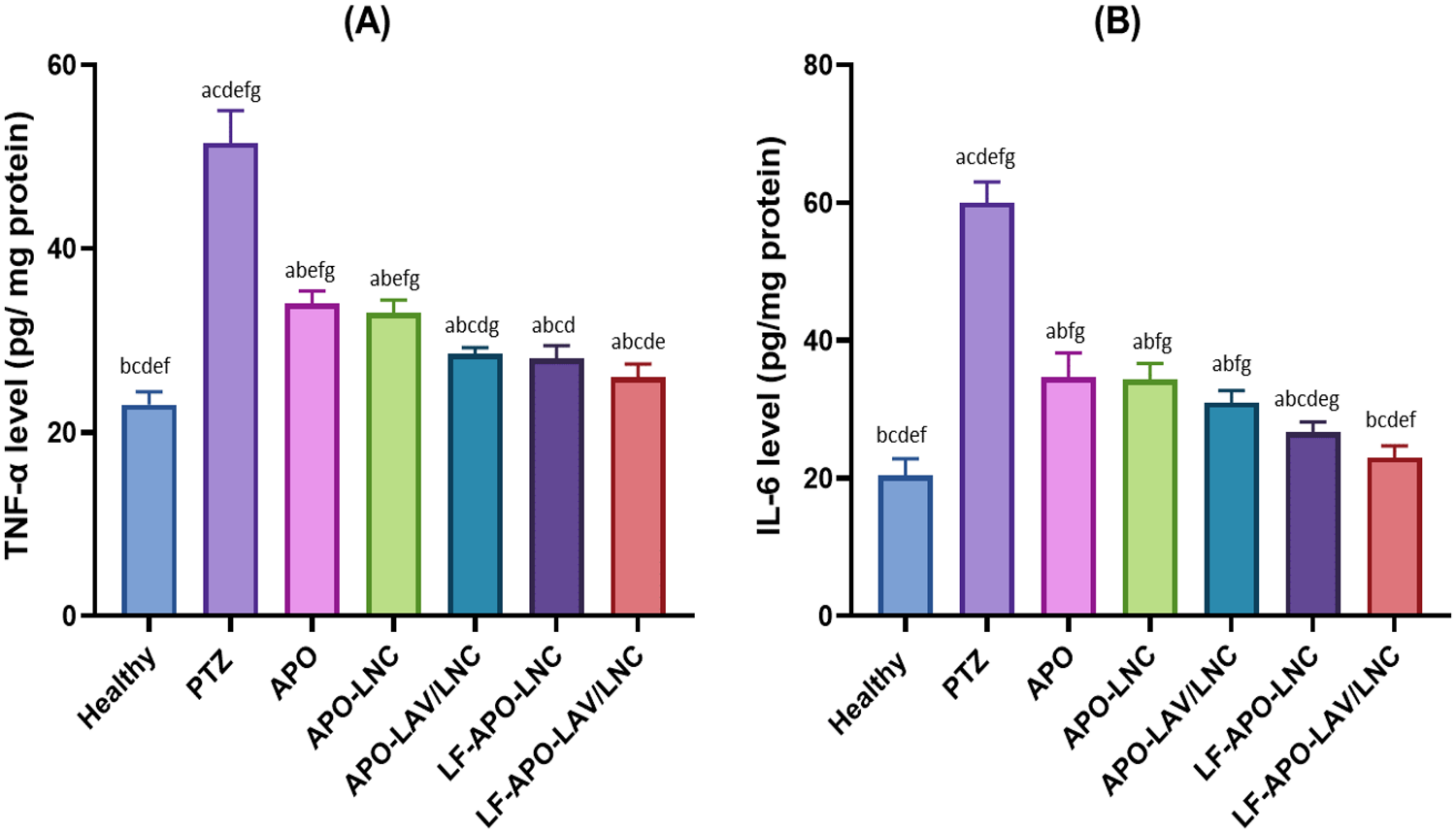
Effect of APO and APO loaded LNC formulations pretreatment on proinflammatory markers: **A** TNF-α and **B** IL-6 in brain tissue homogenate in a PTZ-induced seizures model in rats*. *Data presented as mean ± standard deviation (SD) (n = 6). (^a^p ≤ 0.05 vs healthy control, ^b^p ≤ 0.05 vs positive control (PTZ), ^c^p ≤ 0.05 vs APO, ^d^p ≤ 0.05 vs APO-LNC, ^e^p ≤ 0.05 vs APO-LAV/LNC, ^f^p ≤ 0.05 vs LF-APO-LNC and ^g^p ≤ 0.05 vs LF-APO-LAV/LNC)

Epileptic seizures have been shown to be associated with increased levels of pro-inflammatory cytokines [69] correlating with seizures frequency and duration [92, 93]. The mechanism underlying these increased levels is attributed to the overproduction of ROS which in turn activates other inflammatory regulators as NF-kB and COXII that further stimulate release of proinflammatory mediators [94]. In this study, TNF-α and IL-6 were selected to elucidate the effect of APO and its LNC formulations on modulating inflammatory response following PTZ induced seizures as shown in Fig. 8.

Following PTZ injection a 2.3-fold increase in TNF-α and a threefold increase in IL-6 were observed compared to the healthy group. Pretreatment with APO or APO-LNC formulations brought about a significant reduction in proinflammatory cytokines compared to the PTZ group (p ≤ 0.05) with an insignificant difference between the 2 groups (p > 0.05). Both modification of LNC oily core with LAV (APO-LAV/LNC) and coating with LF (LF-APO-LNC) resulted in further comparable reduction in inflammatory markers (p > 0.05). Remarkably, LF-APO-LAV/LNC achieved a level of both TNF-α and IL-6 tantamount to the healthy control group (p > 0.05). This enhanced effect results from the combined anti-inflammatory action of APO via inhibition of NADPH oxidase regulating proinflammatory mediators [35], LAV [95] through reduction of TNF-α expression and LF suppressing the expression of proinflammatory cytokines [26] besides enhancing permeability across the BBB.

#### Histopathological examination

**Fig. 9:**
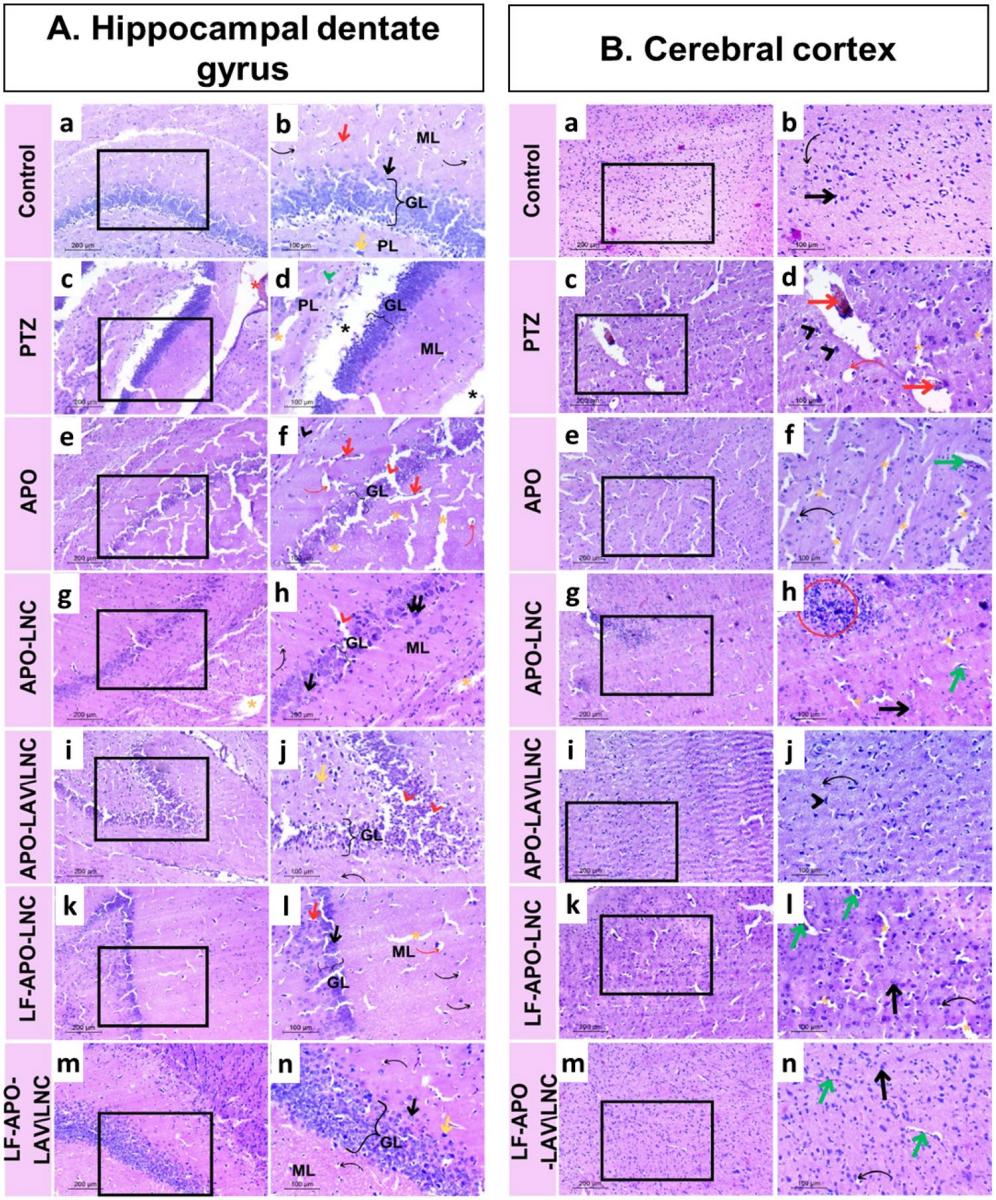
Photomicrographs of H&E-stained brain sections of rats showing **A** hippocampal dentate gyrus (DG) of: (a, b) control group (healthy group) showing normal DG layers: granular layer (GL); polymorphic (PL) and molecular (ML) with normal granule cell (black arrow), normal glial cell (black bent arrow), pyramidal cell (yellow arrow) and normal blood vessel supply (red arrow), (c, d) PTZ (positive control) group showing a thin degenerated granular layer (GL), polymorphic layer (PL), molecular layer (ML) with precellular neuron (green head arrow), hemorrhage (red asterisk), neuropil vacuoles (yellow asterisk) and continuous large vacuoles (black asterisk), (e, f) APO treated group showing a thin granular layer (GL) with vacuoles (red head arrow), congested blood vessels (red arrows), fibrosis (black head arrow); precellular glial cells (red bent arrows) and neuropil vacuolations (yellow asterisks), (g, h) APO-LNC treated group showing a thin granular layer (GL) with normal granule cells (black arrow) and apoptotic neurons (double black arrow) interrupted with vacuole. Glial cells appeared normal (black bent arrow) and neuropil vacuole (yellow asterisk), (i, j) APO-LAV\LNC treated group showing a thick granular layer (GL) with vacuoles (red head arrow) and normal pyramidal cell (yellow asterisk), (k, l) LF-APO-LNC treated group showed a thin granular layer with normal granule cells (black arrow), normal blood vessel (red arrow), neuropil vacuole (yellow asterisk), normal glial cells (black bent arrow), and precellular glial cells (red bent arrow), (m, n) LF-APO-LAV\LNC treated group showing normal thickness of granular (GL) and molecular (ML) layers, normal granule cells (black arrow), glial cells (black bent arrow) and pyramidal cells (yellow arrow) and **B** Cerebral cortex of: (a, b) control group (healthy group) showing normal neuron structure (black arrow) and normal glial cells (black bent arrow), (c, d) PTZ (positive control) group showing hemorrhage (red arrow), pyknotic neurons (black head arrow); abnormal glial cells (red bent arrow), and neuropil vacuoles (yellow asterisk), (e, f) APO-treated group showing congested blood vessels (green arrow), normal glial cells (black bent arrow), and neuropil vacuoles (yellow asterisks). (g, h) APO-LNC-treated group showed inflammation (red circle), normal blood vessels (green arrow), normal neurons (black arrow) and neuropil vacuoles (yellow asterisks). (i, j) APO-LAVLNC-treated group showed abnormal neuron structure (black head arrow) and normal glial cells (black bent arrow). (k, l) LF-APO-LNC-treated group showed congested blood vessels (green arrows), neuropil vacuoles (yellow asterisks), normal neuron (black arrow) and normal glia cells (black bent arrows). (m, n) LF-APO-LAVLNC-treated group showed normal components of the cerebral cortex: blood vessels (green arrow), body neurons (black arrow), and glial cells (black bent arrow). Right panels (magnification X200, scale bar 100 µm) are higher magnifications of the insets of the corresponding left panels (magnification X 100, scale bar 200 µm)

The hippocampal dentate gyrus (DG) is essential to healthy spatial memory and other cognitive activities and hence is implicated in a variety of neurological illnesses and mental disorders, including epilepsy [96]. Also, several studies described the Epileptogenic Zone (EZ) as the area of cortex essential and adequate for initiating seizures [97]. In this context, histopathological features focusing on how epilepsy disrupts the cerebral cortex and hippocampus for evaluation of potential protective benefits of APO or APO LNC formulations pretreatment were examined (Fig. 9).

##### Hippocampus

The healthy group displayed the three DG layers: molecular (ML), granular (GL), and polymorphic (PL) or hilus layers in their usual state. The granular layer was composed of small, tightly packed granule cells with rounded vesicular nuclei. In addition, glial cells are numerous in both ML and PL, along with pyramidal cells that have normal structural integrity (Fig. 9A (a, b)). On the other hand, the positive control group (PTZ group) showed several alterations in the pattern of neuron degeneration, including precellular holes in neurons with neuropil vacuolation or apoptotic neurons. The discontinuity of the DG layers is also reflected in the massive continuous vacuoles linked to the thin granular layer. Hemorrhage was also clearly visible (Fig. 9A (c, d)).

Even though APO therapy demonstrated the absence of massive continuous vacuolations, neuropil vacuoles were seen along the sector. Additionally, ML and PL showed signs of neruoglial cell precelluar holes. The PL layer revealed fibrosis-related characteristics that could be connected to this group's abnormal blood vessels (Fig. 9A (e, f)). Also, APO-LNC showed a reduced average number of granular necrotic neurons since normal neurons begin to develop alongside those necrotic ones. Despite the glial cells' normal appearance, some signs of neuropil vacuolation were still present. (Fig. 9A (g, h). LNC modification with the bioactive oil; LAV increased GL thickness by limiting the presence of apoptotic neurons and increasing normal pyramidal cells in the APO-LAV/LNC treated group (Fig. 9A (i, j)) while LF coating (LF-APO-LNC group) enhanced the structure of granular neurons regardless of the layer thickness with a large number of normal glial cells (Fig. 9A (k, l)). The synergistic consequences of LAV and LF were clearly shown histologically in the LF-APO-LAV\LNC group in terms of normal hippocampal cytoarchitecture, comprising all layer components except for a minimal amount of neuropil vacuolation (Fig. 9A (m, n)).

##### Cerebral cortex

The healthy group showed normal prefrontal cortex with neuronal cell bodies with rounded open face nuclei and distinct nucleoli encircled by a border of basophilic cytoplasm. They also demonstrated normal neuroglial cells. Neuropil had an acidophilic appearance and was entangled with healthy blood capillaries in a web of neuronal and glial cell processes (Fig. 9A (a, b)). In contrast, the positive control (PTZ group) revealed shrunken neural cell bodies with highly pigmented pyknotic nuclei; some of these cells had pointed ends resembling flames. Aberrant glial cells, either in the form of precellular glial cells or separate glial nuclei with dilated hemorrhagic blood capillaries and vacuolated neuropil were also evident (Fig. 9A (c, d)).

Treated groups demonstrated varying degrees of improvement relative to the PTZ group in ways reflecting their individual treatment potentials. First, despite the disappearance of hemorrhagic blood vessels in the APO-treated group, significant neuropil fibrosis and vacuolation were clearly seen (Fig. 9A (e, f)). The APO-LNC treated group showed normal cellular structure with little neuropil vacuolation and no fibrosis as evidence of recovery (Fig. 9A (g, h)).

APO-LAV/LNC treated group showed normal neuron structure but still exhibited aberrant glial organization and irregular background (neuropil) (Fig. 9A (i, j)). In the LF-APO-LNC group, the background color was not uniform, reflecting neuropil deficiency and the formation of vacuolations (Fig. 9A (k, l)). Interestingly, the LF-APO-LAV/LNC group showed histopathological features of the cerebral cortex comparable to the healthy group (Fig. 9A (m, n)).

#### Immunohistochemical assessment

**Fig. 10:**
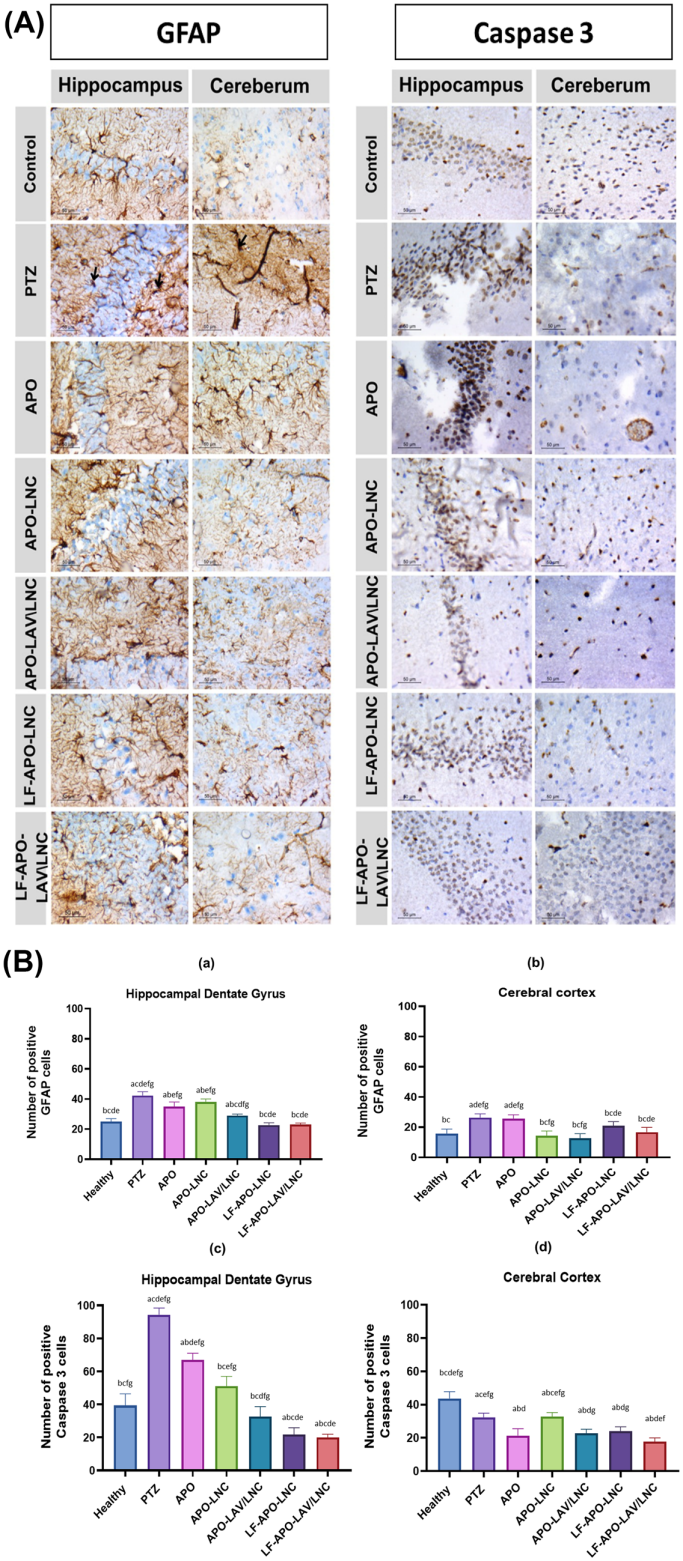
**A** Immunohistochemical demonstration of GFAP and caspase-3 in hippocampal dentate gyrus and cerebral cortex of PTZ induced epileptic rat model pretreated with APO and APO-LNC formulations compared to healthy and PTZ (positive control) groups (magnification × 1000, scale bar 100 µm). Black arrows refer to activated glial cells and **B** Morphometric analysis of GFAP and caspase-3 positive cells in hippocampal dentate gyrus and cerebral cortex. Data presented as mean ± standard deviation (SD) (n = 5). (^a^p ≤ 0.05 vs healthy control, ^b^p ≤ 0.05 vs positive control (PTZ), ^c^p ≤ 0.05 vs APO, ^d^p ≤ 0.05 vs APO-LNC, ^e^p ≤ 0.05 vs APO-LAV/LNC, ^f^p ≤ 0.05 vs LF-APO-LNC and ^g^p ≤ 0.05 vs LF-APO-LAV/LNC)

Immunohistochemical observation supports the histopathological features observed in the H&E stained sections of the hippocampus and cerebral cortex. The effect of different treatment options on the induced epilepsy state was assessed by GFAP and caspase-3 immunostaining (Fig. 10A and B). The expression of these potential cellular stress markers; GFAP and caspase-3 reflect tissue degeneration [97]. Increased GFAP expression denotes the presence of reactive astrocytes and gliosis during neurodegeneration [98]. Apart from the positive control group (PTZ group) and the APO-treated group, GFAP was not expressed in the cerebral cortex when compared to the hippocampal dentate gyrus, showing that the treatment disrupted the progression of this condition to the cerebral cortex. Notably, the appearance of tissue disruption shown in H&E and immunostaining is what accounts for the decline in the number of positive GFAP cells in the PTZ and APO groups at the level of the cerebral cortex. On the other hand, the hippocampus showed GFAP immunopositivity indicating strong gliosis in PTZ when compared to other treatment groups. APO treatment resulted in a statistically significant reduction in GFAP expression compared to the positive control (PTZ) (p ≤ 0.05). Although no significant difference was observed between APO and APO-LNC treated groups (p > 0.05), LAV incorporation in APO-LAV/LNC demonstrated a significant reduction in GFAP expression (p ≤ 0.05) compared to APO and APO-LNC. Moreover, LF-APO-LAV/LNC demonstrated the most successful treatment technique for the immunoreactive region (Fig. 10A and B (a and b)) showing no significant difference compared to normal tissue (p > 0.05).

Considering caspase 3; the increased expression was related to enhancing the neuronal apoptosis by PTZ injection [56] depending on activation of inflammatory pathway. All groups have suboptimal expression of caspase 3 as an apoptotic marker in the cerebral cortex. Notably the treated groups with different APO formulations showed signs of degenerated tissue explaining the reason for the lower expression of this apoptotic marker. Being NADPH oxidase inhibitor, APO reduce ROS production controlling apoptotic pathway [32]. Assessment revealed that all experimental groups had an increase in the number of Caspase-3 positive markers in the hippocampus compared to the cerebral cortex, confirming the presence of acute epilepsy (Fig. 10A and B (c and d)). In hippocampal dentate gyrus, LAV incorporation in APO-LAV/LNC showed no significant difference when compared to healthy group. The highest reduction in caspase-3 compared to PTZ group exhibited by LF-APO-LAV/LNC (4.7-fold) could be attributed to the dual action of LAV [44] and LF in reducing caspase-3 production [95] and inhibiting caspase protease family [26] thus eliminating apoptotic pathway.

## Conclusion

In the current work, LNC for the subcutaneous delivery of APO were developed. For an augmented antiseizure effect, LNC were modified with the bioactive essential oil; LAV. Essential oil incorporation into LNC oily core revealed a remarkable enhancement in antiepileptic action as evaluated biochemically, histopathologically and immunohistochemically. LNC were further coated with LF to improve BBB permeability and brain retention. Efficient coating on selected LNC formulations based on electrostatic interaction between positively charged LF and negatively charged APO-LNC was achieved. LF coating brought about additional improvement in the assessed parameters in addition to increased bioavailability and brain retention. The augmented antiseizure and neuroprotective effect of LF-APO-LAV/LNC relative to APO and the uncoated formulation reflect the modulation of different signaling pathways involved in epileptogenesis via the combinatory action of APO, LAV and LF. These findings present LF-APO-LAV/LNC as a promising brain targeted nanoplatform circumventing epilepsy in vivo and encourage future research on other CNS disorders. Also, the results highlight the potential of herbal drugs, essential oils and nanotechnology in the management of seizures. Further studies are required to show the effect of source and essential oil composition on the nanosystem efficacy.

## Data Availability

The authors confirm that the data for this study 624 findings are available within the article and the supplementary information file.

## Declarations

### Animal studies

All institutional and national guidelines for the care and use of laboratory animals were followed.
